# Understanding the impact of five major determinants of health (genetics, biology, behavior, psychology, society/environment) on type 2 diabetes in U.S. Hispanic/Latino families: Mil Familias - a cohort study

**DOI:** 10.1186/s12902-019-0483-z

**Published:** 2020-01-06

**Authors:** Jessikah Morales, Namino Glantz, Arianna Larez, Wendy Bevier, Mary Conneely, Ludi Fan, Beverly Reed, Carlos Alatorre, Rosirene Paczkowski, Tamim Ahmed, Andrew Mackenzie, Ian Duncan, David Kerr

**Affiliations:** 1 Sansum Diabetes Research Institute, 2219 Bath Street, Santa Barbara, CA 93105 USA; 2 Eli Lilly and Company, Indianapolis, IN USA; 3 Santa Barbara Actuaries, Santa Barbara, CA USA

**Keywords:** Type 2 diabetes, Hispanic Americans, Latino, Social determinants of health

## Abstract

**Background:**

In the United States (U.S.), the prevalence of both diagnosed and undiagnosed type 2 diabetes (T2D) is nearly twice as high among Mexican-origin Hispanic/Latino adults compared to non-Hispanic Whites. Rates of diabetes-related complications, e.g., acute stroke and end-stage renal disease, are also higher among Hispanic/Latino adults compared to their non-Hispanic/Latino White counterparts. Beyond genetic and biological factors, it is now recognized that sociocultural influences are also important factors in determining risk for T2D and the associated complications. These influences include ethnicity, acculturation, residence, education, and economic status. The primary objective of this study is to determine the influence of the 5 major determinants of human health (genetics, biology, behavior, psychology, society/environment) on the burden of T2D for Latino families*.* To achieve this objective, Mil Familias (www.milfamilias.sansum.org/) is establishing an observational cohort of 1000 Latino families, with at least one family member living with T2D.

**Methods:**

Specially trained, bilingual Latino/a community health workers (Especialistas) recruit participant families and conduct research activities. Each individual family member will contribute data annually on over 100 different variables relating to their genetics, biology, psychology, behavior, and society/environment, creating a Latino-focused biobank (“Living Information Bank”). This observational cohort study is cross-sectional and longitudinal. Participants are divided into 4 groups: adults age ≥ 18 years with and without T2D, and children age ≥ 7 and < 18 years with and without T2D. Study activities take place through encounters between families and their Especialista. Encounters include screening/enrollment, informed consent, health promotion assessment, laboratory tests, questionnaires, physical activity monitoring, and reflection.

**Discussion:**

By creating and providing the framework for the Cohort Establishment study, we intend to inform new approaches regarding equity and excellence in diabetes research and care. We will examine the complex set of factors that contribute to the burden of diabetes in Latino families and assess if cardio-metabolic disease risks go beyond the traditional biological and genetic factors. Breaking the code on the interplay of cardio-metabolic risk factors may help not only this fast growing segment of the U.S. population, but also other high-risk populations.

**Trial registration:**

Study retrospectively registered at ClinicalTrials.gov (NCT03830840), 2/5/2019 (enrollment began 2/1/2019).

## Background

In the United States (U.S.), members of racial and ethnic minority groups are disproportionally affected by type 2 diabetes (T2D). For example, the prevalence of both diagnosed and undiagnosed T2D is nearly twice as high among Mexican-origin Hispanic/Latino adults compared to non-Hispanic Whites [1]. Furthermore, the American Heart Association has reported that only 14% of adult Hispanic/Latino Americans meet the criteria for “ideal cardiovascular health” as compared to 20% for non-Hispanic/Latino White Americans [2]. Rates of diabetes-related complications including premature death from diabetes, acute stroke and end-stage renal disease are also higher among Hispanic/Latino adults compared to their non-Hispanic/Latino White counterparts [3].

Beyond genetics and biological factors, such as achieved hemoglobin A1_c_ (HbA_1c_) levels, lipids, obesity and blood pressure, it is now recognized increasingly that sociocultural influences are also important factors in determining an individual’s risk related to the development of T2D and the associated complications. These influences include ethnicity (e.g., Mexican-Americans have a greater diabetes burden than Cuban-Americans); duration and impact of acculturation (the process by which immigrants adopt the attitudes, values, customs, beliefs, and behaviors of a new culture); place of residence; achieved education level; and economic status [4, 5]. Compounding this burden, Hispanic/Latinos are also a minority among diabetes care providers, researchers, or research participants. For example, Hispanic/Latinos represent just 1% of clinical trial participants, and Hispanic/Latinos are a small fraction of providers (e.g., Hispanic/Latinos are about 40% of California’s population but only 8% of nurses and 5% of doctors) [6, 7]. Therefore, given the previous lack of Hispanic/Latino participation in clinical research, this study aims to (a) find novel approaches to engage this population and (b) consider psychological, behavioral and environmental factors, as well as the traditional biological and genetic influences on the risk of progression of T2D in this growing but underserved U.S. population, as these have not be undertaken previously.

## Methods/design

In this study, we acknowledge the use of the term “Hispanic/Latino” when referring to individuals because they identify as persons of Latin American descent who live in the U.S. or Latin America and trace their origin to countries in the Americas where the predominant languages are Spanish, Portuguese, and French [4]. But henceforth we refer to study participants as “Latino(s)” to be inclusive of participants of Latin American origin who do not speak Spanish.

### Aims

*The primary objective of this study is to determine the influence of the 5 major determinants of human health (genetics, biology, behavior, psychology, society/environment) on the real-world burden of T2D for Latino families.* To achieve this objective, beginning February 2019, Mil Familias (www.milfamilias.sansum.org/) began the establishment of an observational cohort of 1000 Latino families with at least one family member currently living with T2D, among whom we are studying the impact over time of the 5 main determinants of human health. We work with specially trained, bilingual Latino/a community health workers (known as Especialistas) who recruit participant families, conduct research activities including data collection, and, when necessary, refer families to appropriate community resources. Facilitated by these Especialistas, each individual family member contributes data annually on over 100 different variables relating to their genetics, biology, psychology, behavior, and society/environment, creating a Latino-focused biobank (“Living Information Bank”). Once the cohort is established, data analyses will inform the development of evidence-based, collaborative, and culturally-relevant prevention and treatment strategies.

An operational pilot involving over 100 adult Latino individuals with T2D, completed in 2018, established the feasibility of methods and created the infrastructure to scale to the 1000-family cohort (Mil Familias-Santa Barbara’s Operational Pilot to Understand Diabetes in the Latino Community NCT03736486).

### Primary outcome

The primary outcome of Mil Familias cohort establishment is the creation of an observational cohort of 1000 Latino families with at least one family member living with T2D, by deploying the methods proven feasible by Mil Familias Operational Pilot. The Mil Familias Cohort Establishment will expand upon the Mil Familias Operational Pilot cohort and infrastructure to collect and store the up to 100 proposed variables across the 5 determinants of human health (genetics, biology, behavior, psychology and the environment) to support long-term Mil Familias implementation. The variables pertaining to each participant are collected and stored during an initial visit series and then annually for up to 3 years.

### Major secondary outcomes

These include (a) achieved HbA_1c_ levels; (b) cardio-metabolic risk including blood pressure, waist circumference, and insulin sensitivity; (c) food security; and (d) activity levels. Details of additional secondary end-points have been published previously (https://clinicaltrials.gov/ct2/show/NCT03830840).

In addition to the variables listed above, we also collect prospective data related to major cardiovascular events (3-point MACE: cardiovascular death, non-fatal myocardial infarction and non-fatal stroke). Data sources include participants and their families, collaborating health centers, and the local provider of in-hospital clinical care. At enrollment, participants provide written, informed consent for Mil Familias researchers to request data from the relevant provider organization’s electronic medical record system on reported clinical events. Additional information are collected on new onset diabetes including gestational diabetes, unscheduled attendances at emergency rooms, and hospital admissions due to uncontrolled diabetes.

### Design

**Fig. 1:**
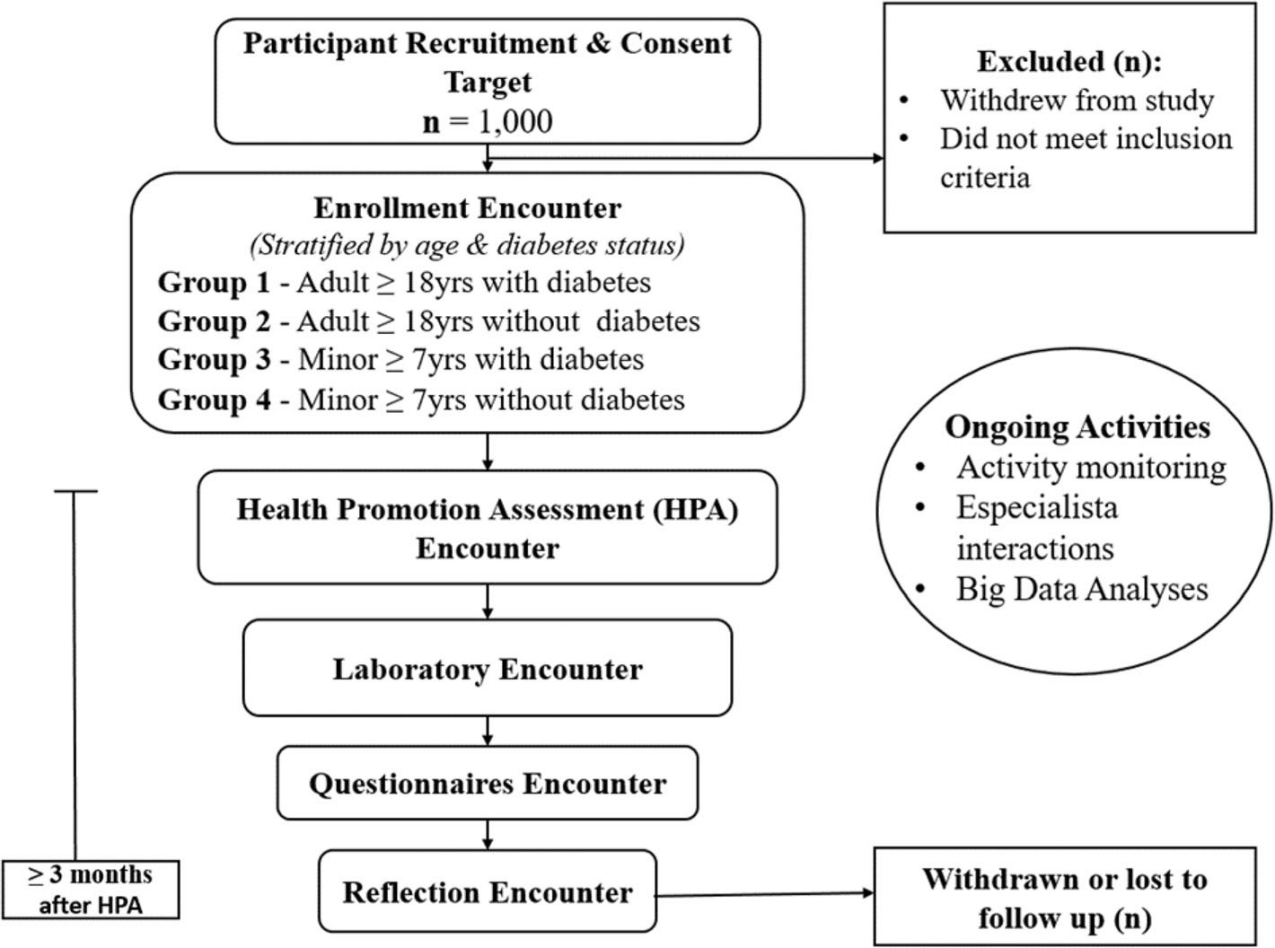
Study design. Encounters may be split or combined as long as the Reflection encounter occurs at least 3 months after the HPA encounter

The study is an observational cohort trial that is cross-sectional and longitudinal. Participants are divided into groups by age and diabetes status (described below). The overall design is shown in Fig. 1.

### Setting and participants

In Santa Barbara County, the population is 45.6% Latino, most of Mexican ancestry [8]. To establish the Mil Familias cohort, up to 1200 families may be screened and enrolled with an estimated 20% screen-fail or dropout rate, resulting in approximately 1000 families in the study. Potential participants are selected from Santa Barbara County and surrounding communities or satellite sites with the help of existing community allies and healthcare organizations. Approaches to recruitment include engagement with and referrals from: local Federally Qualified Health Centers (FQHCs); community service providers; academic institutions with high English as a Second Language enrollment (i.e., Hispanic Serving Institutions); local employers; local media (Spanish and English language); social media; advertisements; participation in community events; SDRI physical site and websites; and ad hoc interaction with Especialistas. This type of combined approach was successful in the operational pilot. Only participants who meet all eligibility criteria are enrolled and study staff confirm participant eligibility during the screening and enrollment encounter. A “family” is followed as one unit, but is estimated to include on average 3 to 5 individuals (based on findings from the operational pilot). Therefore, up to 6000 individuals may be screened and enrolled.

The definition of “Mil Familias family” accounts for statistical, tracking, and reporting requirements of this study and is based on the U.S. Census definition of a “family group” and modified to include single-person units and relatedness via domestic partnership [9]. Each individual may participate in just one Mil Familias family. A “Mil Familias family” consists of an enrolled individual family representative and any enrolled family members. The family representative is the first enrolled. A family member is any individual subsequently enrolled who: (a) resides with the family representative (i.e., co-resides) and (b) is an immediate relative (spouse/partner, parent, child, or sibling) to the family representative by birth, marriage/domestic partnership, or adoption.

**Inclusion Criteria** (family representative)

- Males or females ≥ 7 years of age at enrollment.
- Self-reported Hispanic/Latino heritage.
- Co-resides with immediate family member (as defined above) or self with established diagnosis of T2D.
- Signed and dated written informed consent by the date of enrollment.
- Based on the research staff’s judgment, participant or participant’s representative must have a good understanding, ability, and willingness to adhere to the protocol, including performance of self-monitored data collection during the wearable device portion of the study.

**Inclusion Criteria** (family member)

- Males or females ≥ 7 years of age at enrollment.
- Self-reported Hispanic/Latino heritage.
- Co-resides with immediate family representative.
- Signed and dated written informed consent by the date of enrollment.
- Based on the research staff’s judgment, participant or participant’s representative must have a good understanding, ability, and willingness to adhere to the protocol, including performance of self-monitored data collection during the wearable device portion of the study.

**Exclusion Criteria**

- Life expectancy < 6 months.
- Any active clinically significant physical or mental disease or disorder, which in the investigator’s opinion could interfere with the participation in the study.
- Language barriers precluding comprehension of study activities and informed consent.
- Participation in other research studies involving medication or device within 1 month prior to enrollment.
- Known or suspected abuse of alcohol, narcotics, or illicit drugs.

**Participant Groups**

Individual participants are assigned to a data collection group stratified by age and T2D status, as follows. The group serves to identify the type of data collected for that individual.

- Group 1 consists of adults aged ≥ 18 years with T2D.
- Group 2 consists of adults aged ≥ 18 years without T2D.
- Group 3 consists of children aged ≥ 7 years and < 18 years with T2D.
- Group 4 consists of children aged ≥ 7 years and < 18 years with T2D without T2D

### Especialistas

The Mil Familias Operational Pilot proved the feasibility of Sansum Diabetes Research Institute’s (SDRI’s) “Especialista” model, in which bilingual Latino/a community health workers are trained by SDRI in diabetes identification, research methodology, and referrals to healthcare and other social needs resources, and then employed to conduct research.

Upon enrollment, each family is paired with an Especialista who speaks their preferred language/s (Spanish and/or English). Individuals meet face-to-face with their Especialista as well as have access via email or phone to their Especialista while enrolled in the study. Especialistas facilitate research activities, which include administering the informed consent and conducting study encounters. The encounters involve delivering questionnaires, conducting interviews, and training on wearable devices. Especialistas may facilitate participant connections to research, education, and care opportunities relevant to preventing and managing diabetes, and address barriers to accessing such resources by serving as a trusted community member contact and liaison with research staff. The research team meets with the Especialistas on a weekly basis for debriefing on study progress as well as for continuing education.

### Enrollment

Enrollment begins with the informed consent process. Participant eligibility is confirmed following review of the inclusion and exclusion criteria by study staff during the Enrollment Encounter. Each participant is provided with oral and written information (English and/or Spanish) describing the nature and duration of the study. Prior to initiation of any study-related procedures, the participant signs and dates a written informed consent to participate in the study, including the California Experimental Subject’s Bill of Rights and Health Insurance Portability and Accountability authorization forms. These forms are retained with SDRI records, and each participant receives a copy. For minor participants (aged ≥7 years and < 18 years), both participant and parent receive study information; the participant’s parent signs on behalf of the minor participant and the minor signs an assent form.

The participant is assigned and given contact information for their Especialista who is their point of contact for Mil Familias. An emergency telephone number is also provided, as well as the SDRI contact information for non-urgent assistance during normal business hours. Participants are instructed to contact the research staff if they experience any adverse events or untoward occurrences while enrolled in the study. Participants are provided with a unique study identifier. They are asked to bring this identifier with them to all Mil Familias encounters.

After the informed consent process, data collection begins, including administration of a brief introductory interview and collection of basic sociodemographic information. The Especialistas schedule the participant’s next visit.

### Study activity series and scheduling encounters

Study activities take place through face-to-face interaction between families and study staff; these visits are referred to as “encounters.” Encounters are organized into “series,” with activities varying based on whether the series is for a new participant or a continuing participant. Each new participant completes an initial encounter series, including screening/enrollment and informed consent, Health Promotion Assessment (HPA), laboratory tests, questionnaires, and reflection, as well as physical activity monitoring and interaction with the Especialista with whom they are paired. After having been enrolled in the study for at least 12 months, participants are eligible to start the continuing participant encounter series. The continuing participant encounter series involves activities similar to the new participant encounter series. While continuing participation is preferred, it is not required to be eligible for the study.

In the course of this 3-year protocol, participants will complete at least 1 new participant encounter series and, possibly, 0, 1, or 2 continuing participant encounter series, depending on their initial enrollment date. Regardless of series type (new participant or continuing participant), participants have the flexibility to complete encounters in accordance with their personal schedules as long as encounter series are completed in chronological order. The last encounter, Reflection, in a 12-month participation cycle occurs at least 3 months after the HPA encounter.

Families may attend study encounters as a group, but individual data collection and informed consent occurs one-on-one in a private setting, unless the participant is a minor. As detailed above, participants are assigned to a data collection group based on their age and diabetes status.

### Health promotion assessment (HPA) and biological specimen collection encounter

Height, weight, and waist circumference are measured following the guidelines in the National Health and Nutrition Examination Survey Anthropometry Procedure Manual, January 2016 [10]. Body mass index is then be calculated. Blood pressure is measured following 2017 guidelines set forth by the American College of Cardiology/American Heart Association Task force on Clinical Practice Guidelines [11]. A foot screening is performed by trained Especialistas with medical provider guidance.

In addition, participants meet with qualified, trained study staff and, depending on age, diabetes status, and personal preferences, the following biological specimens may be collected:

- Epithelial cell sample via cheek swab to collect genetic material [DNA Genotek, Ontario, Canada, or Salimetrics, Carlsbad, CA, USA]
- Saliva sample to collect genetic material [DNA Genotek, Ontario, Canada or Salimetrics, Carlsbad, CA, USA]
- Urine sample to assess protein levels and potential kidney damage (laboratory screening sample)*
- Stool sample to analyze bacteria lining the digestive tract for microbiome genomic testing [DNA Genotek, Ontario, Canada or Fecal Occult Blood Test, Beckman Coulter, Brea, CA, USA]
- Finger-stick HbA_1c_ test [Siemens DCA Vantage, Siemens Healthcare, Norwood, MA, USA]

*Note: Urine samples may be collected at the HPA or Laboratory Encounter.

### Laboratory encounter

Participants meet with trained study staff, (e.g. licensed phlebotomist) for laboratory tests that may include:

- Urine sample to assess protein levels and potential kidney damage (laboratory screening sample)*
- Fasting venous blood draw, which is analyzed immediately for complete blood count (no differential) panel, HbA1c, comprehensive metabolic panel, lipid panel, thyroid panel, C-peptide, C-reactive protein quantitative, and insulin, and other samples are transported to the Mil Familias biobank for processing, storage, and future analysis.

The blood drawing limit is determined for all participants based on the participant’s age, weight, and blood draw history; volume per draw is determined based on NIH Guidelines for Clinical Research Purposes [12], and in consultation with the Principal Investigator if needed.

Blood collection is done in a fasting state (at least 8 h), unless the participant takes insulin. Only certified personnel perform blood collection. Blood is drawn into evacuated blood collection tubes for the requested laboratory tests and for biobank storage. One 10 ml red top and one 10 ml lavender top with EDTA is drawn with the tubes inverted 6–8 times. The EDTA lavender top tube is then centrifuged (2000 g for 20 min) as soon as possible after collection. Serum red top tubes sit at room temperature for at least 30 min and not longer than 2 h before centrifugation. Biobank samples are processed within 4 h of the draw. Plasma, serum, and buffy coat or leukocytes are separated into sterile storage cryotubes, identification labels are attached, and samples are placed in a − 80 °C freezer with constant remote temperature monitoring.

### Questionnaires

Questionnaires are completed by select Mil Familias participants depending on age, diabetes status, and whether the participant is a new or continuing participant. This encounter may take place either at SDRI, a partner medical clinic, or at a community location. The Especialista verbally administers and, if necessary, assists with the participant-reported questionnaires. Questionnaires for participants include topics that cover the 5 major determinants of health: genetics, biology, behavior, psychology, and society/environment (Additional file 1).

### Activity monitoring

Participants aged 13 years and over are asked to wear wearable activity monitor/s each day for up to a week and up to twice within a 12-month period, with at least 6 months between each wear period. The purpose of this stratification is to capture activity during the spring-summer season versus the fall-winter season.

The Especialista and/or research staff dispense the wearable device/s to the participant and train them on how to use the wearables. Participants wear a Fitbit Charge 2™ (Fitbit, Inc., San Francisco, CA) (or a similar Fitbit activity tracker) on the non-dominant wrist and an ActiGraph wGT3X-BT (ActiGraph, LLC, Pensacola, FL) monitor on the dominant hip. Participants are asked to wear the Fitbit at night, and the ActiGraph can be removed at night and replaced in the morning. After dispensing the device/s, each participant or their representative fills in an activity questionnaire to distinguish physical activity relating to the participant’s occupation from physical activity via leisure-time exercise. Participants are asked to return the wearable device/s after one week. Both devices are then downloaded to a computer at the study center; Fitbits are not wirelessly synchronized to a mobile phone application. Adherence is defined as ≥12 h/day of wear time for at least 3 of the 7 days, and the 3 days of wear was chosen to represent approximately half of the possible wear time. Upon return of wearable device/s, each participant or their representative may complete an Activity Monitor Follow-up Survey.

### Reflection

Depending on age and diabetes status, participants may complete a brief questionnaire regarding their Mil Familias experience and an additional finger stick HbA_1c_ may be measured. Additionally, participants may be invited to attend a focus group on their Cohort Establishment study experience and recommendations.

### Data collection, data storage, and statistical analysis

#### Determination of sample size

Up to 1200 families will be screened in the Mil Familias Cohort Establishment study. Given anticipated participation of Latino/a people with T2D as well as their family members, up to 6000 participants may eventually form the Mil Familias cohort. Participants are recruited in multiple places and via a variety of methods, resulting in a diverse and heterogeneous cohort.

#### Data collection

A list of proposed variables/measures to be collected during the Mil Familias Cohort Establishment study can be found in Table 1 and Table 2. In the tables:

- *Child* refers to participants aged ≥ 7 and < 18 years; adult refers to participants ≥ 18 years.
- *Par* refers to the parent/guardian providing information on behalf of the participating child.
- *Opt* refers to items that are optional, i.e., not required.
- Administration and use of patient-reported questionnaires are subject to copyright and translation timelines and may be excluded in order to proceed.

**Table 1:** Intended variables for data collection

Variable	Encounter and/or Intended Tool or Method (Ref.)	1 – Adult with diabetes	2 – Adult w/o diabetes	3 – Child with diabetes	4 – Child w/o diabetes
CHILDREN/YOUTH VARIABLES FOR ALL 5 DETERMINANTS OF HEALTH					
All variables in minors	NSCH: National Survey Children’s Health [13]	–	–	Par	Par
GENETIC INFLUENCES					
Biobank samples					
Blood	Plasma, serum, buffy coat collected via venous draw	Yes	Yes	Opt	Opt
Epithelial cells	Collected via cheek swab	Yes	Yes	Opt	Opt
Saliva	2 ml saliva into vial collected via spitting into a cup	Yes	Yes	Opt	Opt
Microbiome	Stool sample collected at home and mailed to lab	Opt	Opt	Opt	Opt
BIOLOGICAL INFLUENCES					
Family medical history	HPA: Health Promotion Assessment	Yes	Yes	Par	Par
Physiological markers					
HbA_1c_	Blood draw and fingerstick	Yes	Yes	Opt	Opt
Metabolic panel	Blood draw and urinalysis	Yes	Yes	Opt	Opt
Lipid panel	Blood draw	Yes	Yes	Opt	Opt
Vital signs					
Body mass index (BMI)	HPA: Health Promotion Assessment	Yes	Yes	Yes	Yes
Waist circumference	HPA: Health Promotion Assessment	Yes	Yes	Yes	Yes
Blood pressure	HPA: Health Promotion Assessment	Yes	Yes	Yes	–
Heart rate and rhythm	HPA: Health Promotion Assessment	Yes	Yes	–	–
Foot screening	HPA: Health Promotion Assessment	Yes	–	Yes	–
Date of last dental exam	HPA: Health Promotion Assessment	Yes	Yes	Par	Par
Date of last eye exam	HPA: Health Promotion Assessment	Yes	–	Par	–
Neuropathy (Y/N)	HPA: Health Promotion Assessment	Yes	–	Par	–
Retinopathy (Y/N)	HPA: Health Promotion Assessment	Yes	–	Par	–
History					
Medical history	Medical history, including date of diabetes diagnosis	Yes	Yes	Par	Par
Medication history	Medical history and visual inspection of medications	Yes	Yes	Par	Par
Most recent flu shot	Intro. Interview	Yes	Yes	Par	Par
DM-related foot disease	Q-DFD: Quest. for Diabetes-Related Foot Disease [14]	Yes	–	–	–
Women only					
Menopausal status	HPA: Health Promotion Assessment	Yes	Yes	–	–
Pregnancy status	HPA: Health Promotion Assessment	Yes	Yes	–	–
Number of children	HPA: Health Promotion Assessment	Yes	Yes	–	–
Birthweights of children	HPA: Health Promotion Assessment	Yes	Yes	–	–
Type 1 DM in children	HPA: Health Promotion Assessment	Yes	Yes	–	–
Gestational DM	HPA: Health Promotion Assessment	Yes	Yes	–	–
PSYCHOLOGICAL INFLUENCES					
Quality of life	SF-12: Health Survey Short Form [15]	Yes	Yes	–	–
Adverse childhood events	ACES: Adverse Childhood Events Questionnaire [16]	Yes	Yes	–	–
Depression	PHQ-9: Patient Health Questionnaire [17]	Yes	Yes	–	–
Ethnic discrimination	PEDQ-CV: Perceived Ethnic Discrimination Quest [18].	Yes	Yes	–	–
Spirituality	FACIT-Sp-12: Spiritual Well-Being Scale [19]	Yes	Yes	–	–
Diabetes risk perception	RPS-DD: Risk Perception Survey-Developing Diabetes [20]	–	Yes	–	–

*Abbreviations*: *DM* Diabetes mellitus, *HbA*_*1c*_ Hemoglobin A_1c_, *Intro* Introductory, *Opt* Optional, *Par* Participant

**Table 2:** Intended variables for data collection, continued

Variable	Encounter and/or Intended Tool or Method (Ref.)	1 – Adult with diabetes	2 – Adult w/o diabetes	3 – Child with diabetes	4 – Child w/o diabetes
BEHAVIORAL INFLUENCES					
Sleep					
Patient-reported	OSQ: Oviedo Sleep Questionnaire [21]	Yes	Yes	–	–
Device-reported	Wearable device (e.g., Fitbit)	Yes	Yes	≥ 13	≥ 13
Physical activity					
Patient-reported	Activity Log	Yes	Yes	–	–
Device-reported	Wearable device (e.g., Fitbit, ActiGraph)	Yes	Yes	≥ 13	≥ 13
Diet					
Tortilla consumption	Intro. Interview, # corn vs. flour eaten daily past 30 days	Yes	Yes	–	–
Sugary drinks	Intro. Interview, BRFSS questions [22]	Yes	Yes	–	–
Eating out	Intro. Interview, NHANES question [23]	Yes	Yes	–	–
Stress	PSS-4: Perceived Stress Scale [24]	Yes	Yes	–	–
Drug abuse	DAST-10: Drug Abuse Screening Test [25]	Yes	Yes	–	–
DM management	DSSCI: Diabetes Symptom Self-Care Inventory [26]	Yes	–	–	–
Family DM management	FSE: Family Self-Efficacy for Diabetes Management [27]	–	Yes	–	–
Foot care	FCCS-FCB: Foot Care Confidence Scale / Behavior [28]	Yes	–	–	–
SOCIAL-ENVIRONMENTAL INFLUENCES					
Socio-demographics					
Age	Intro. Interview/Demographics	Yes	Yes	Par	Par
Gender	Intro. Interview/Demographics	Yes	Yes	Par	Par
Race/ethnicity	Intro. Interview/Demographics	Yes	Yes	Par	Par
Address	Intro. Interview/Demographics/Contacts	Yes	Yes	Par	Par
Phone number	Intro. Interview/Demographics/Contacts	Yes	Yes	–	–
Email	Intro. Interview/Demographics/Contacts	Yes	Yes	–	–
Household size, income	Intro. Interview/Demographics	Yes	Yes	Par	Par
Education	Intro. Interview/Demographics	Yes	Yes	Par	Par
Insurance status	Intro. Interview/Demographics	Yes	Yes	Par	Par
Insurance type/payer	Intro. Interview/Demographics	Yes	Yes	Par	Par
Recent provider visit	Intro. Interview/Demographics	Yes	Yes	Par	Par
Country of birth	Intro. Interview/Demographics	Yes	Yes	Par	Par
Marital status	Intro. Interview/Demographics	Yes	Yes	–	–
Occupation	Intro. Interview/Demographics	Yes	Yes	–	–
Night shifts	Intro. Interview/Demographics	Yes	Yes	–	–
Alcohol, tobacco use	Intro. Interview/Demographics	Yes	Yes	–	–
Acculturation	BASH: Brief Acculturation Scale for Hispanics [29]	Yes	Yes	–	–
Introductory Interview					
DM cause	Intro. Interview: Why is DM common in Latino families?	Yes	Yes	–	–
DM prevention	Intro. Interview: What can prevent DM in Latino families?	Yes	Yes	–	–
DM challenges	Intro. Interview: What difficulties to get help with DM?	Yes	Yes	–	–
Health literacy	SAHL: Short Assessment of Health Literacy [30]	Yes	Yes	–	–
Health numeracy	NVS: Newest Vital Signs [31]	Yes	Yes	–	–
Trust in physician	TPS: Trust in Physician Scale [32]	Yes	Yes	–	–
Social needs stability	SNST: Health Leads Social Needs Screening Toolkit [33]	Yes	Yes	–	–
Food (In)security	FSSM: Food Security Survey Module [34]	Yes	Yes	–	–
Functional support	SSQ6: Social Support Questionnaire short version [35]	Yes	–	–	–
Support structure	mMOS-SSS: Modified Medical Outcomes Study Social Support Survey [36]	Yes	Yes	–	–

*Abbreviations*: *DM* Diabetes mellitus, *HbA*_*1c*_ Hemoglobin A_1c_, *Intro*., Introductory, *Opt* Optional, *Par* Participant

#### Data storage

REDCap Cloud (www.redcapcloud.com), a secure Part 11 Validated Electronic Data Capture application, is managed by trained study staff at SDRI and used to hold all records and sociodemographic, biometric, psychosocial, and wearable participant data. Data may be collected in the following ways: recorded on hard copy or electronic source documents by study staff and Especialistas, securely extracted from partnering medical clinic Electronic Health Record system, and self-collected by participants via wearable devices. Study participant identity is protected by assigning participants a unique identifier upon enrollment. All data used in the analysis and reporting is de-identified and will not contain any Protected Health Information. No identifiable patient information will be released. Access to the cloud-based Mil Familias database is restricted to those SDRI researchers who have been granted permission by the Mil Familias research team.

Participants who drop out during the screening are recorded as screening failures in the participant’s file and no further follow-up is required. The data is included in the study database and is reported. An individual participant is to be withdrawn from the study if the participant withdraws consent for study participation, without the need to justify the decision. All data is included in the study database and is reported.

#### Data analysis

Statistical analyses will be performed in collaboration with SDRI’s data partner, Santa Barbara Actuaries, Inc. All statistics will be considered significant with *p* values < 0.05. Chi squared tests will be conducted to detect differences in categorical outcomes. T-tests will be conducted to test differences in continuous outcomes where the sample size is less than 50 for any sub population. Z tests will be conducted for continuous variables with a sample size greater than 50 where there is no evidence of violation of normality. For continuous outcomes that may violate the normality assumption, a log-normal transformation or other appropriate analysis may be applied.

In addition, independent variables may be confounders of each other’s effect on the outcomes and thus consideration will be given to explore likely confounders. There are adjustment approaches, such as propensity score matched cohorts, to address such biases. Regression analysis for a particular outcome can also be performed on various individual characteristics.

Based on the experience from the Operational Pilot, we expect the sample size of the full study to be sufficient to provide substantial levels of statistical significance to compare the relationship between outcomes and individual variables. For example, based on the Operational Pilot (*n* = 107), HbA_1c_ can be stratified into 2 groups (> 9 and < 7%). In the Pilot, we found that 40% had an HbA_1c_ < 7%, and for 28% it was > 9%. This was significant at the 3% level and this will fall below 1% with 160 participants and below 0.1% at 280. Our full study population is designed to have 1000 members, so we believe that the majority of individual variables of interest will be powered to show statistically significant conclusions.

### Quality control of data collection

In concordance with the Mil Familias Operational Pilot Study, standard operating procedures (SOPs) are developed or adapted and followed to ensure standardized implementation of the study protocol. In addition, training modules for the Especialistas and other study staff are created and implemented using structured templates. More specifically, these trainings focus on (but are not limited to): recruitment, Good Clinical Practice (GCP), T2D knowledge, care navigation, data collection and management. Further, a mandatory three-person data verification check is in place from the initial data collection through final data input into a secure electronic data capture system (REDCap EDC System). Given the high retention rate (over 85% of participants completed all study visits) and associated completeness of individual and aggregate datasets in the Mil Familias Operational Pilot Study, it is anticipated that this study will achieve similar sustained levels of data quantity and quality, contributing to its overall success.

## Discussion

Currently, Latinos bear an unacceptable excess and unfair burden of cardio-metabolic disease and the associated serious complications. This Cohort Establishment study will create a large observational cohort of 1000 Latino families impacted by T2D. Data collected from participant families will be used to create a unique, Latino-focused Living Information Bank which will help determine future effective evidence-based prevention and treatment intervention strategies that are equitable and culturally-relevant.

Public health programs have utilized community health workers (CHWs) with much success [37–40]. Growing research demonstrates the positive impact of CHW efforts and the significant cost savings they generate. Findings confirm that CHWs are highly effective at providing instruction on health care issues such as nutrition and physical activity, particularly for Latino patients who have diabetes, and this results in significantly improved diabetes control and health outcomes [41]. By using Especialistas in this study, we anticipate effective outreach, participation, and retention with our study participants.

Based on the success of the initial Mil Familias Operational Pilot study, we anticipate that the Cohort Establishment study will be completed in the proposed time period. The participating Latino families are given the opportunity to build trust-based relationships with their Especialistas who act as their personal health liaison and advocate throughout the study. Such an exchange of trust is vital to the data collection process as the aim is to create a safe-space in which participants feel comfortable to share private and personal information. The uniqueness of this study is centered on collecting comprehensive cross-sectional and longitudinal information from a traditionally hard-to-reach population.

### Limitations

While a potential limitation of this study is lack of an active control group, there are other cohorts of interest published elsewhere, including the Hispanic Community Health Study/Study of Latinos (HCHS/SOL). This is a community-based cohort study of 16,415 self-identified Hispanic/Latino adults of which 2148 individuals self-reported a diagnosis of diabetes [42]. Additionally, it is worth noting that the study population could be limited to individuals of Mexican heritage by excluding from analysis Latino individuals from other countries of origin, such as Panamanians, Hondurans, or Guatemalans.

By creating and providing the framework for the Cohort Establishment study, we intend to inform new approaches regarding equity and excellence in diabetes research and care. In taking a deeper look into the complex set of factors that contribute to the burden of diabetes in Latino families, we will assess if cardio-metabolic disease risks go beyond the traditional biological and genetic factors. In this pursuit, further investigation regarding the factors that affect the Latino population will help to determine and promote novel prevention and intervention strategies to reduce the burden of cardio-metabolic disease in this population. Breaking the code on the interplay of cardio-metabolic risk factors may very well help not only this fast growing segment of the U.S. population, but also other high-risk populations in the U.S. and around the globe.

## Supplementary information

**Additional file 1.** Introductory Interview and Demographics. Activity Monitor Survey. Reflection Questionnaire.

## Data Availability

The SDRI-developed study-specific questionnaires (Introductory Interview and Demographics, Activity Monitor Survey, Reflection Questionnaire) have been uploaded as additional files. References and/or links to all other study materials are provided in the References section (Additional file 1).

## Abbreviations

CHW: Community health worker
FQHC: Federally Qualified Health Center
HbA_1c_: Hemoglobin A_1c_
HPA: Health Promotion Assessment
SDRI: Sansum Diabetes Research Institute
T2D: Type 2 diabetes
U.S.: United States

## References

[1] National Center for Health Statistics (US). Health, United States, 2016: With Chartbook on Long-term Trends in Health. Hyattsville (MD): National Center for Health Statistics (US); 2017 May. Available from: https://www.ncbi.nlm.nih.gov/books/NBK453378/28910066

[2] Mozaffarian D, Benjamin EJ, Go AS, Arnett DK, Blaha MJ, Cushman M (2016). Heart disease and stroke Statistics-2016 update: a report from the American Heart Association. Circulation..

[3] Golden SH, Brown A, Cauley JA, Chin MH, Gary-Webb TL, Kim C (2012). Health disparities in endocrine disorders: biological, clinical, and nonclinical factors--an Endocrine Society scientific statement. J Clin Endocrinol Metab.

[4] Aviles-Santa ML, Colon-Ramos U, Lindberg NM, Mattei J, Pasquel FJ, Perez CM (2017). From Sea to Shining Sea and the Great Plains to Patagonia: A Review on Current Knowledge of Diabetes Mellitus in Hispanics/Latinos in the US and Latin America. Front Endocrinol (Lausanne).

[5] Beydoun MA, Beydoun HA, Mode N, Dore GA, Canas JA, Eid SM (2016). Racial disparities in adult all-cause and cause-specific mortality among us adults: mediating and moderating factors. BMC Public Health.

[6] Coakley M, Fadiran EO, Parrish LJ, Griffith RA, Weiss E, Carter C (2012). Dialogues on diversifying clinical trials: successful strategies for engaging women and minorities in clinical trials. J Women's Health (Larchmt).

[7] Ibarra A (2018). In a diverse state, California’s Latino doctors push for more of their own Latinx Physicians of California January.

[8] United States Census Bureau. Quick facts Santa Barbara County, California. https://www.census.gov/quickfacts/fact/table/santabarbaracountycalifornia/RHI725217#RHI725217. Accessed 11 April 2019.

[9] United States Census Bureau. Families and Households. https://www.census.gov/topics/families/families-and-households/about/glossary.html. Accessed 11 April 2019.

[10] National Center for Health Statistics (2017). National Health and Nutrition Examination Survey (NHANES) Anthropometry Procedures Manual. Centers for Disease Control.

[11] Whelton PK, Carey RM, Aronow WS, Casey DE, Collins KJ, Dennison Himmelfarb C (2018). 2017 ACC/AHA/AAPA/ABC/ACPM/AGS/APhA/ASH/ASPC/NMA/PCNA guideline for the prevention, detection, evaluation, and Management of High Blood Pressure in adults: executive summary: a report of the American College of Cardiology/American Heart Association task force on clinical practice guidelines. Circulation..

[12] National Institutes of Health (2009). Guidelines for Limits of Blood Drawn for Research Purposes in the Clinical Center. Policy and Communications Bulletin, The Clinical Center.

[13] Child and Adolescent Health Measurement Initiative (2017). National Survey of Children’s Health, Sampling and Survey Administration. Health Resources and Services Administration (HRSA) MaCHBM.

[14] Bergin SM, Brand CA, Colman PG, Campbell DA (2009). A questionnaire for determining prevalence of diabetes related foot disease (Q-DFD): construction and validation. J Foot Ankle Res.

[15] National Center for Health Statistics (US). Health, United States, 2016: With Chartbook on Long-term Trends in Health. Hyattsville (MD): National Center for Health Statistics (US); 2017 May. Available from: https://www.ncbi.nlm.nih.gov/books/NBK453378/. 28910066

[16] Johns Hopkins School Of Public Health. ACEs Resource Packet: Adverse Childhood Experiences (ACEs) Basics. https://www.childhealthdata.org/docs/default-source/cahmi/aces-resource-packet_all-pages_12_06-16112336f3c0266255aab2ff00001023b1.pdf. .

[17] Kroenke K, Spitzer RL, Williams JB (2001). The PHQ-9: validity of a brief depression severity measure. J Gen Intern Med.

[18] Brondolo E, Kelly KP, Coakley V, Gordon T, Thompson S, Levy E (2005). The perceived ethnic discrimination questionnaire: development and preliminary validation of a community version. J Appl Soc Psychol.

[19] Bredle J, Salsman JM, Debb SM, Arnold BJ, Cella D (2011). Spiritual well-being as a component of health-related quality of life: the functional assessment of chronic illness therapy—spiritual well-being scale (FACIT-Sp). Religions..

[20] RPS-DD (2019). Risk Perception Survey for Developing Diabetes. Regents of the University of Michigan.

[21] Paz Garcia-Portilla M, Saiz PA, Diaz-Mesa EM, Fonseca E, Arrojo M, Sierra P (2009). Psychometric performance of the Oviedo sleep questionnaire in patients with severe mental disorder. Rev Psiquiatr Salud Ment.

[22] Centers for Disease Control and Prevention (CDC) (2017). Behavioral Risk Factor Surveillance System Survey Questionnaire. Atlanta, Georgia: U.S. Department of Health and Human Services.

[23] National Health and Nutrition Examination Survey (NHANES) 2017–2018 - Diet Behavior and Nutrition DBQ.895 G/Q [Internet]. U.S. Department of Health and Human Services. 2017–2018. https://wwwn.cdc.gov/nchs/nhanes/continuousnhanes/questionnaires.aspx?BeginYear=2017. Accessed 23 May 2019.

[24] Cohen S, Kamarck T, Mermelstein R (1983). A global measure of perceived stress. J Health Soc Behav.

[25] Skinner HA (1982). The drug abuse screening test. Addict Behav.

[26] Garcia AA (2011). The diabetes symptom self-care inventory: development and psychometric testing with Mexican Americans. J Pain Symptom Manag.

[27] McEwen MM, Pasvogel A, Murdaugh CL (2016). Family self-efficacy for diabetes management: psychometric testing. J Nurs Meas.

[28] Garcia-Inzunza JA, Valles-Medina AM, Munoz FA, Delgadillo-Ramos G, Compean-Ortiz LG (2015). Validity of the Mexican version of the combined foot care confidence / foot-care behavior scale for diabetes. Rev Panam Salud Publica.

[29] Mills SD, Malcarne VL, Fox RS, Sadler GR (2014). Psychometric evaluation of the brief acculturation scale for Hispanics. Hisp J Behav Sci.

[30] Lee SY, Stucky BD, Lee JY, Rozier RG, Bender DE (2010). Short assessment of health literacy-Spanish and English: a comparable test of health literacy for Spanish and English speakers. Health Serv Res.

[31] Singh R, Coyne LS, Wallace LS (2015). Brief screening items to identify spanish-speaking adults with limited health literacy and numeracy skills. BMC Health Serv Res.

[32] Anderson LA, Dedrick RF (1990). Development of the Trust in Physician scale: a measure to assess interpersonal trust in patient-physician relationships. Psychol Rep.

[33] Leads H (2018). Health Leads screening toolkit.

[34] US Department of Agriculture Economic Research Service (2012). U.S. Household Food Security Survey Module: Six-Item Short Form.

[35] Sarason IG, Sarason BR, Shearin EN, Pierce GR (1987). A brief measure of social support: practical and theoretical implications. J Soc Pers Relat.

[36] Moser A, Stuck AE, Silliman RA, Ganz PA, Clough-Gorr KM (2012). The eight-item modified medical outcomes study social support survey: psychometric evaluation showed excellent performance. J Clin Epidemiol.

[37] Kane EP, Collinsworth AW, Schmidt KL, Brown RM, Snead CA, Barnes SA (2016). Improving diabetes care and outcomes with community health workers. Fam Pract.

[38] Tang TS, Funnell M, Sinco B, Piatt G, Palmisano G, Spencer MS (2014). Comparative effectiveness of peer leaders and community health workers in diabetes self-management support: results of a randomized controlled trial. Diabetes Care.

[39] Shah M, Kaselitz E, Heisler M (2013). The role of community health workers in diabetes: update on current literature. Curr Diab Rep.

[40] Spencer MS, Kieffer EC, Sinco B, Piatt G, Palmisano G, Hawkins J (2018). Outcomes at 18 months from a community health worker and peer leader diabetes self-management program for Latino adults. Diabetes Care.

[41] Egbujie BA, Delobelle PA, Levitt N, Puoane T, Sanders D, van Wyk B (2018). Role of community health workers in type 2 diabetes mellitus self-managemnt: a scoping review. PLoS One.

[42] Casagrande SS, Aviles-Santa L, Corsino L (2017). Hemoglobin A1c, blood pressure, and Ldl-cholesterol control among Hispanic/Latino adults with diabetes: results from the Hispanic community health study/study of Latinos (Hchs/sol). Endocr Pract.

