# Supplementary material for: Understanding the impact of five major determinants of health (genetics, biology, behavior, psychology, society/environment) on type 2 diabetes in U.S. Hispanic/Latino families: Mil Familias - a cohort study
Source: BMC Endocr Disord. 2020 Jan 6;20:4. doi: 10.1186/s12902-019-0483-z (PMC6945604; doi:10.1186/s12902-019-0483-z)
Supplement: Supplementary file 1 — Additional file 1. Introductory Interview and Demographics. Activity Monitor Survey. Reflection Questionnaire. [file 12902_2019_483_MOESM1_ESM.pdf]

Mil Familias Reflection Survey

Instructions for Especialista:

The purpose of this questionnaire is to learn from the participant about what worked well in Mil Familias Cohort Establishment and what can be improved for the future.

For questions 1-3, read the question aloud to the participant, but do NOT read the response options. If the participant does not have a ready answer, THEN read ALL the response options to the participant. Based on the participant's response, check the boxes that most closely correspond with their answer. You may check all answers that apply.

1. Which part/s of Mil Familias (if any) made the biggest impact on your health? (check all that apply)
   - ◻ Working with an Especialista
   - ◻ Receiving lab test results
   - ◻ Getting a health promotion assessment
   - ◻ Getting referred to a local community resource
   - ◻ Using the activity watch and/or belt
   - ◻ Other ____
   - ◻ Other ____
   - ◻ Mil Familias did not make an impact on my health

   Briefly describe any checked:____

   ____

2. Was there anything that made it hard for you to participate in Mil Familias? (check all that apply)
   - ◻ Discomfort with Especialista interaction
   - ◻ Discomfort with lab testing
   - ◻ Discomfort with storing biological samples
   - ◻ Discomfort with health promotion assessment
   - ◻ Discomfort with questionnaires
   - ◻ Treatment by clinic staff
   - ◻ Talking about sensitive information
   - ◻ Transportation to and from appointments
   - ◻ Scheduling visits around work responsibilities
   - ◻ Scheduling visits around home / family responsibilities
   - ◻ Childcare
   - ◻ Other______________________________
   - ◻ Other ______________________________
   - ◻ There was nothing that made it hard for me to participate in Mil Familias

   Briefly describe any checked:____

   ____

3. Was there anything that made it easy for you to participate in Mil Familias?
   - ◻ Being part of learning more about diabetes among Latino families
   - ◻ Working with an Especialista
   - ◻ Receiving lab test results
   - ◻ Getting a health promotion assessment
   - ◻ Getting referred to a local community resource
   - ◻ Using an activity watch and/or belt
   - ◻ Other ____
   - ◻ Other ____
   - ◻ There was nothing that made it easy for me to participate in Mil Familias

   Briefly describe any checked:____

   ____

Instructions for Especialista:<br><br>**For questions 4-5**, read the following questions AND the response options to the participant.

4. Mil Familias is going to continue into the future, with you (if you want) and new people participating.

   Suppose that you were in charge of Mil Familias and could make TWO changes that would make the program better for participants like you. What would you change (**select two**)?
   - ◻ More interaction with your Especialista
   - ◻ Free childcare available during visits
   - ◻ Free transportation to/from visits
   - ◻ Visit location closer to home or worksite
   - ◻ Keep the activity watch or belt
   - ◻ Visits scheduled on weekends or evenings
   - ◻ Get an in-depth explanation on what the lab tests mean
   - ◻ Other ____
   - ◻ Other ____
   - ◻ I would not change anything about Mil Familias

5. What part/s would you keep the same (**select two**)?

   - ◻ Interaction with Especialista
   - ◻ Receiving lab tests
   - ◻ Having a health promotion assessment
   - ◻ Being referred to local community resources
   - ◻ Using the activity watch and/or belt
   - ◻ Other ____
   - ◻ Other ____

6. Were the questionnaires easy to understand?
   - ◻ Very easy
   - ◻ Somewhat easy
   - ◻ Somewhat difficult
   - ◻ Difficult

7. The main goal of this project is to learn more about diabetes in Latino families. Is there anything that we didn't ask you during Mil Familias, that you think we should understand about diabetes in Latino families?

   ____

   ____

   ____

8. If you were inviting a friend or relative to participate in this project, what would you say in the invitation?

   ____

   ____

   ____
